# 4-Hydroxynonenal, a Potential Biomarker for Lung Inflammatory Diseases

**DOI:** 10.3390/ijms27083366

**Published:** 2026-04-09

**Authors:** Nancy Kaushal, Alexandria K. Vo, Nathan C. Kobus, Riddhi B. Dave, Kota V. Ramana

**Affiliations:** Department of Biomedical Sciences, Noorda College of Osteopathic Medicine, Provo, UT 84606, USA

**Keywords:** oxidative stress, hydroxynonenal, asthma, COPD, ARDS, lungs

## Abstract

Asthma, chronic obstructive pulmonary disease (COPD), and acute respiratory distress syndrome (ARDS) are the major lung inflammatory complications affecting the global population. Exposure to allergens, infections, smoking, and environmental pollutants could cause persistent oxidative stress and dysregulated immune responses, leading to lung inflammatory complications. Increased oxidative stress can lead to lipid peroxidation and the formation of toxic lipid aldehydes. One of the major lipid aldehydes formed during lipid peroxidation is 4-hydroxy-2-nonenal (4-HNE). 4-HNE is well known to covalently modify proteins, nucleic acids, and lipids, thus modifying cellular signaling pathways and inflammatory cascades. Increased levels of 4-HNE have been identified in lung tissues, bronchoalveolar lavage (BAL) fluid, and the serum of patients with inflammatory lung conditions. Further, 4-HNE contributes to airway remodeling, mitochondrial dysfunction, and modulation of inflammatory responses in the lung epithelial cells. Recent studies also indicate the potential role of 4-HNE as an important mediator and a potential biomarker of various human disease progression, including the diagnosis and monitoring of lung inflammatory diseases. In this narrative review, we discuss current evidence on the pathological role of 4-HNE, its potential as a biomarker, and its importance for early detection and for potential therapeutic strategies in lung inflammatory complications.

## 1. Introduction

Lung inflammatory diseases, such as chronic obstructive pulmonary disease (COPD), asthma, and acute respiratory distress syndrome (ARDS), remain among the leading causes of morbidity and mortality worldwide. Infections, allergens, smoking, and pollutants are the major causes of these complications. For example, the recently ended COVID-19 pandemic, where the major cause of death in patients is viral pathogen-induced severe lung inflammation, which leads to ARDS [1]. Several allergens, such as ragweed, tree pollen, mold, and dust mites, could cause severe complications of asthma and COPD [2,3]. Further, cigarette smoking, auto and industrial smoke, and pollutants could trigger chronic bronchitis and emphysema, leading to COPD [4,5]. In addition, environmental exposures and genetic predisposition involving certain genes have been associated with the development of asthma and COPD, especially at the early onset of disease progression. These pollutants, allergens, and infections can cause oxidative stress, disrupt redox homeostasis, and lead to immune dysregulation. Increased oxidative stress has been shown to be a primary driver of chronic immune and inflammatory responses, as well as tissue dysfunction and damage. Furthermore, oxidative stress has been shown to be a major cause of several human diseases, such as inflammatory complications, infections, cardiovascular complications, hyperglycemia, neurodegenerative diseases, and cancer [6,7,8,9]. Increased oxidative stress generates various reactive oxygen species (ROS), which can alter various cellular metabolic and signaling pathways and cause tissue dysfunction and damage. Further, oxidative stress has been shown to increase NF-κB signalosome-induced generation of pro-inflammatory cytokines, chemokines, and growth factors, which, in an autocrine and paracrine manner, further increase the inflammatory response and organ dysfunction. Additionally, oxidative stress can also activate the NLRP3 inflammasome. Activation of the inflammasome leads to caspase-1-dependent production of innate immune cytokines such as IL-1β and 1l-18. These cytokines trigger an innate immune response. Thus, smoking, infections, and pollutants make our body’s immune and redox balance weak, leading to various disease complications.

Furthermore, oxidative stress-generated ROS has been shown to cause peroxidation of polyunsaturated fatty acids in the membranes and cause lipid peroxidation [10]. During this process, several secondary toxic lipid aldehydes are generated, including 4-hydroxy -nonenal (4-HNE, 4-hydroxy-2-nonenal), malondialdehyde (MDA), and acrolein. Among these aldehydes, 4-HNE is the most abundant and toxic lipid aldehyde generated during lipid peroxidation [11]. It is highly reactive due to its unsaturated bonds and can spontaneously interact with cellular proteins, lipids, and nucleic acids, as well as alter their structure and function. This can lead to tissue dysfunction and damage. Since lungs are very vulnerable to oxidative stress, we are constantly exposed to airborne allergens, pollutants, and infectious agents, as well as smoke and other chemicals. These oxidants could increase ROS production, reduce antioxidant defenses, and promote oxidative and inflammatory responses. Further, increased formation of lipid peroxidation-derived aldehydes could further damage the cellular and metabolic signaling pathways in the airway linings and lung epithelial cells, leading to airway remodeling, mucous production, and chronic inflammation [12,13].

**Figure 1:**
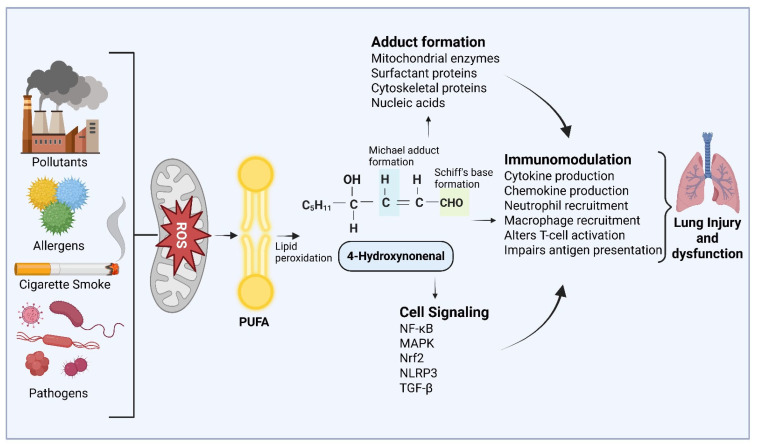
Significance of 4-HNE in oxidant-induced lung injury. Environmental pollutants, cigarette smoke, infections, and allergens cause oxidative stress and increase the production of reactive oxygen species (ROS). Free radical attack on membrane polyunsaturated fatty acids (PUFAs) leads to lipid peroxidation and the accumulation of 4-hydroxynonenal (4-HNE). Further, 4-HNE through Michael addition and Schiff’s base formation forms covalent adducts with proteins and nucleic acids spontaneously. These adducts have been shown to impair mitochondrial function and interfere with cellular barriers. Further, 4-HNE modulates inflammatory signaling pathways, promotes immune cell activation and cytokine release, leading to lung injury and dysfunction. The image was created in BioRender. COM, N. (2026), https://BioRender.com/pt5yfpa (accessed on 28 February 2026).

Several studies indicate that cigarette smoke could trigger lipid peroxidation and generate 4-HNE, leading to COPD [14]. In asthma, increased airway hyperresponsiveness and remodeling are associated with increased 4-HNE levels [15]. Allergens such as ragweed have been shown to increase eosinophilic inflammation and increase NADPH oxidative activity, which can generate ROS and generate 4-HNE, leading to asthma complications [16]. Similarly, bacterial and viral infections can generate ROS and significant 4-HNE adducts, which promote epithelial barrier disruption and fibrosis in ARDS [17]. These studies suggest that 4-HNE could play a major role in the progression of lung inflammatory complications (Figure 1).

In addition, 4-HNE has been shown to modify proteins involved in redox signaling, energy metabolism, and immune response [11]. For example, 4-HNE has been shown to activate redox-sensitive transcription factors such as NF-κB and AP-1, leading to the expression of pro-inflammatory cytokines, chemokines, and growth factors [18]. Furthermore, 4-HNE has been shown to inhibit mitochondrial function by binding to electron transport chain complexes, which aggravate ROS production and oxidative damage [19]. It can also induce apoptosis by activating caspases, causing epithelial and endothelial cell loss in lung tissue [20]. It can modulate the expression and activation of antioxidant enzymes, such as heme oxygenase, catalase, and superoxide dismutase, by regulating the Nrf-2 transcription factor. However, the concentrations of HNE are very important in the regulation of Nrf-2. At low concentrations, it can activate, while at high concentrations, it can inhibit Nrf2 [21,22]. A few clinical studies have shown that elevated levels of 4-HNE are present in bronchoalveolar lavage fluid (BAL), exhaled breath condensates, plasma, and lung biopsies from patients with COPD, asthma, and ARDS [11]. The results of these studies indicate that elevated 4-HNE levels may be associated with reduced lung function and disease progression, suggesting that 4-HNE could serve as a prognostic marker.

Further, several studies have shown that targeting the aldose reductase (AR) and aldehyde dehydrogenase (ALDH) enzymes that metabolize 4-HNE could be a potential treatment for lung inflammatory complications. In this narrative review, we discuss the significance of 4-HNE in various lung inflammatory diseases, specifically asthma, COPD, and ARDS. Generally, in asthma, COPD, ARDS, and pneumonitis, inflammation plays a primary role in their development and symptoms, and several studies indicate the significance of 4-HNE in their pathophysiology. Specifically, exposure to allergens in asthma, cigarette smoke in COPD, severe infection in ARDS, and toxins in pneumonitis can activate macrophages, neutrophils, and lymphocytes. These cells then release inflammatory mediators (cytokines, chemokines and leukotrienes), causing airway hyperresponsiveness, mucus production, and alveolar damage. These processes result in airflow limitation, impaired gas exchange and reduced pulmonary function. Although the type and duration of inflammation differ (reversible in asthma, chronic in COPD, acute and severe in ARDS, and interstitial in pneumonitis), they all share the underlying mechanism in which inflammation leads to structural and functional impairment of the lungs, justifying their classification as inflammatory lung complications [23]. Further, we aim to provide readers with a clear understanding of the pathological role of 4-HNE in these complications and to describe potential therapeutic strategies to modulate 4-HNE metabolism. PubMed searches have been conducted for the past decade or so using keywords oxidative stress, 4-HNE, asthma, COPD, ARDS, and lung inflammatory complications. We have included only empirical studies, peer-reviewed studies, and journal publications. We did not include articles from magazines, commentaries, or any research on other respiratory complications.

## 2. Oxidative Stress, Lipid Peroxidation, and Formation of 4-Hydroxynonenal

Our bodies are constantly exposed to various oxidants, such as airborne particles, pathogens, and environmental pollutants. The skin, eyes, and lungs are always at risk from these oxidants. The exposure of these oxidants generates highly reactive free radical oxygen species such as superoxide anion, hydrogen peroxide, and hydroxyl radicals. Under normal conditions, free radicals are neutralized by antioxidant enzymes, such as superoxide dismutase (SOD), catalase, and glutathione peroxidase, as well as glutathione (GSH). The cellular redox balance enables low levels of ROS to participate in key signaling pathways and maintain cellular homeostasis. Further, low levels of ROS also participate in key signaling pathways, including host defense, cell differentiation, and proliferation. However, during oxidative stress, ROS production overwhelms antioxidant defenses, leading to macromolecular damage and altering oxidative and inflammatory signaling. Further, in the lungs, oxidative stress initiates in the airways and spreads to pulmonary tissues. Generally, oxidative stress can arise from both exogenous and endogenous sources. Exogenous factors, such as cigarette smoke, air pollution, and occupational exposure to dust or chemicals, can increase oxidative stress and elevate free radical levels. On the other hand, endogenous sources of oxidative stress could include activated inflammatory cells (neutrophils and macrophages) in response to pathogen attack, which release ROS and reactive nitrogen species (RNS) via NADPH oxidase and inducible nitric oxide synthase. Mitochondrial dysfunction and endoplasmic reticulum stress in airway epithelial cells can increase ROS production. Thus, an increased oxidative milieu not only damages lung tissue directly but also sustains and amplifies inflammatory responses and affects other organs.

One of the most common effects of oxidative stress is the peroxidation of membrane lipids, which can generate several toxic aldehydes such as 4-HNE and MDA. These lipid peroxidation-derived aldehyde products can form covalent adducts with proteins and nucleic acids [24]. Further modification of proteins by nitration or carbonylation could impair cytoskeletal integrity and receptor signaling [25,26,27]. Similarly, these aldehydes can cause oxidative damage to mitochondrial DNA and mitochondrial dysfunction, leading to tissue damage and dysfunction. They can activate redox-sensitive transcription factors, which can cause increased inflammatory response, leukocyte adhesion, and immune cell modulation [11].

**Figure 2:**
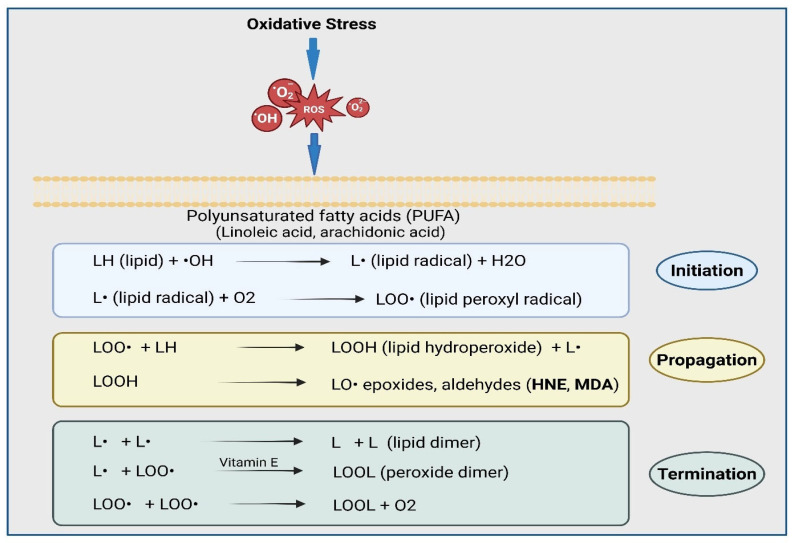
Oxidative stress-induced lipid peroxidation and formation of 4-HNE. Oxidative stress generated reactive oxygen species (ROS), such as hydroxyl radicals (•OH) and superoxide (•O_2_^–^), and hydrogen peroxide (•O_2_^−^) can initiate lipid peroxidation in membrane polyunsaturated fatty acids (PUFAs). ω-6 PUFAs such as linoleic acid and arachidonic acid are particularly susceptible to hydrogen removal due to methylene-interrupted double bonds that create weak C–H bonds. During the initiation phase, the hydroxyl radical abstracts a hydrogen atom from a PUFA to form a lipid radical (L•). Then, the lipid radical reacts with oxygen to generate a lipid peroxyl radical (LOO•). In the next propagation phase, lipid peroxyl radicals abstract a hydrogen atom from an adjacent lipid, forming lipid hydroperoxides (LOOH). This step continues as a chain reaction. During this step, the decomposition of LOOH yields various reactive aldehydes, including malondialdehyde (MDA), acrolein, and 4-HNE. In the termination phase, radical dimerization and vitamin E-mediated hydrogen donation terminate the reaction. The image was created by using BioRender.com. COM, N. (2026), https://BioRender.com/cjwnke4. (accessed on 28 February 2026).

Further oxidative stress leads to lipid peroxidation and the formation of a number of lipid aldehyde signaling intermediates. Free radicals (such as hydroxyl radicals (•OH), superoxide (•O_2_–), and hydrogen peroxide (H_2_O_2_)) can spontaneously attack membrane polyunsaturated fatty acids (PUFAs) and cause lipid peroxidation. Further, specific types of PUFAs, such as linoleic acid, arachidonic acid, and docosahexaenoic acid, are highly susceptible to free radical attack due to the presence of methylene-interrupted double bonds, which confer weak C–H bonds adjacent to double bonds. There are generally three major stages in lipid peroxidation: (1) initiation, (2) propagation, and (3) termination (Figure 2). During the initiation step, hydroxyl radicals abstract a hydrogen atom from the methylene group of PUFAs, resulting in the formation of reactive lipid radicals (L•). These lipid radicals react rapidly with molecular oxygen, resulting in the formation of lipid peroxyl radicals (LOO•). In the next propagation step, the lipid peroxyl radical abstracts a hydrogen atom from an adjacent PUFAs. This step forms lipid hydroperoxides (LOOH) and new lipid radicals that extend the chain reaction. This chain reaction leads to the accumulation of LOOH molecules within cellular membranes. In the termination step, two lipid peroxy radicals (LOO•) react to form a tetroxide (LOO-OOL). Antioxidants, such as vitamin E, donate a hydrogen atom (H•) to a lipid radical (L• or LOO•) and neutralize the lipid radical, forming a stable lipid molecule (LH or LOOH) and a much less reactive antioxidant radical. During this step, as products of termination, a variety of secondary lipid products, such as malondialdehyde (MDA), acrolein, and 4-HNE, are formed. Most importantly, 4-HNE has been shown to be generated specifically from the oxidative fragmentation of ω-6 PUFAs, such as linoleic acid and arachidonic acid [28]. When LOOH molecules undergo homolytic or enzymatic decomposition, β-scission reactions generate reactive α,β-unsaturated aldehydes, among which 4-HNE is the most abundant and biologically active. 4-HNE is a highly electrophilic molecule with both aldehyde and alkene functional groups. This combined reactivity enables HNE to form the Michael adducts or Schiff bases with nucleophilic residues (cysteine, histidine, lysine) in proteins, as well as interact with nucleic acids and membrane lipids.

**Figure 3:**
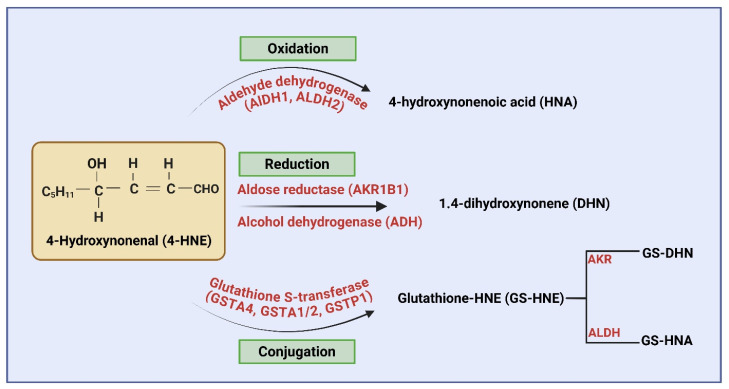
Enzymatic pathways involved in the metabolism of 4-hydroxynonenal (4-HNE). Major enzymatic pathways involved in the metabolism of 4-HNE include oxidation by aldehyde dehydrogenases (ALDH) to form HNA. Reduction by aldose reductase (AKR) and alcohol dehydrogenase (ADH) to DHN and conjugation with glutathione is mediated by glutathione S-transferases. The GS-HNE conjugates are further metabolized by AKR and ALDH to form GS-DHN and GS-HNA, respectively. These conjugates are exported via multidrug resistance proteins (MRP1 and 2) and further processed for biliary and urinary excretion. The image was created by using BioRender.com. COM, N. (2026), https://BioRender.com/mdx372h (accessed on 28 February 2026).

Further, several studies have shown that 4-HNE has a relatively longer half-life compared to free radicals [28]. It can diffuse from its site of origin, exert effects at distant cellular locations, modify several important cellular signaling pathways, and cause cytotoxicity [29]. 4-HNE is highly reactive, forms conjugates with cellular glutathione spontaneously, and is mediated by specific glutathione S-transferases (GSTs) ([30], Figure 3). 4-HNE and its glutathione conjugate (GS-HNE) can be further metabolized by enzymes such as aldose reductase (AR, AKR1B1) and aldehyde dehydrogenases (ALDH2, AKR1C1). AR reduces 4-HNE to 1,4-dihydroxy-2-nonene (DHN) and GS-HNE to GS-DHN [31,32]. Similarly, ALDH2 oxidizes 4-HNE to 4-hydroxy-2-nonenoic acid (HNA) and GS-HNE to GS-HNA [31,33]. In addition, 4-HNE and GS-HNE have been shown to activate pro-inflammatory mediators and induce inflammatory responses [34]. Furthermore, the accumulation of 4-HNE and 4-HNE adducts can contribute to impaired enzyme activity, mitochondrial dysfunction, and inflammatory response, all of which are involved in the pathophysiology of inflammatory diseases, including lung inflammatory complications discussed below [11].

## 3. Hydroxynonenal in Asthma

Asthma is a condition in which the airways become inflamed, narrow, and swell, producing extra mucus that makes it hard to breathe. This can be caused by asthma triggers such as allergies, air pollution, and other airborne irritants [35]. Asthma affects ~5–16% of people worldwide [36]. Most people who have asthma see their first symptoms before the age of 5, but asthma can manifest at any age. Asthma also has a strong genetic component, and several genes have been identified that increase the risk of the disease [37,38]. Current treatments for asthma include bronchodilators and steroid medications [39]. These medications are short-acting beta agonists that provide quick relief during an asthma attack. The most common side effects of prescribed inhalers, such as albuterol, include nervousness, shakiness, headaches, throat and nasal irritation, and muscle aches [40]. Steroid inhalers, such as fluticasone, can cause a sore throat and mouth, a hoarse and croaky voice, coughing, and oral thrush [41]. Other treatments for asthma include decreasing exposure to environmental allergens, such as cockroaches and dust mites, and thermoplasty. Thermoplasty is usually used to treat patients with severe asthma and involves controlled thermal energy applied in consecutive sessions through a bronchoscope to relax the bronchial muscles and improve lung function [42].

The mechanism in asthma begins when mast cells are triggered by an allergic response. Allergens increase cellular oxidative stress and generate ROS. Further, mast cells are activated by an inappropriate immune response to allergens. The antigen binds to dendritic cells, which process and present it to naive T lymphocytes. This triggers one of 2 immune cascades: helper T lymphocyte 1 or helper T lymphocyte 2. If IL-12 is present, Th1 cells are activated. Th1 signals CD8 cell-mediated immunity and the release of neutrophil-mediated cytotoxic inflammatory mediators [43]. This causes the release of tumor necrosis factor and interferon gamma. In the absence of IL-12, the Th2 cytokine cascade is released from CD4 cells, which in turn stimulates IL-5 release. IL-5 recruits eosinophils to the lung tissue, and these cells release proinflammatory interleukins and cytokines that lead to bronchial remodeling. This upregulates inflammatory processes and signals bronchiole contraction, leading to an asthma attack [44].

**Figure 4:**
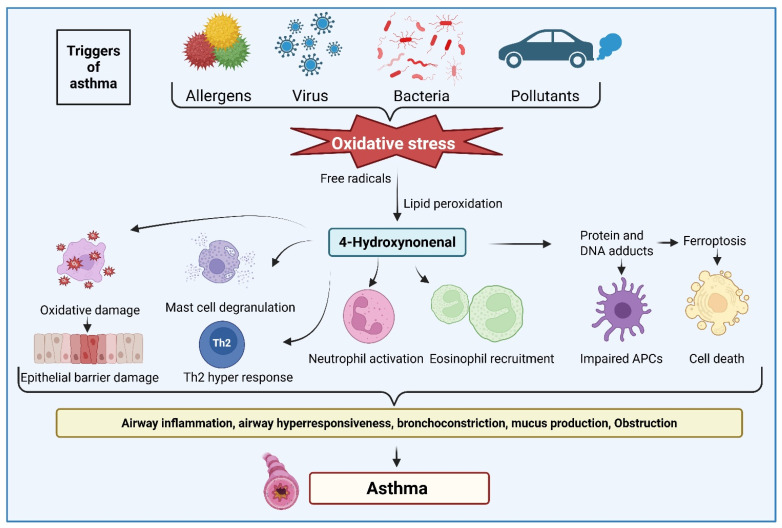
Significance of 4-hydroxynonenal (4-HNE) in the pathophysiology of asthma. Allergens, pollutants, and pathogenic oxidants cause oxidative stress and generate 4-HNE. Elevated 4-HNE accumulates in airway tissues and forms covalent adducts with proteins, and promotes epithelial damage, mast cell degranulation, Th2 hyperresponsiveness, and recruitment of neutrophils and eosinophils. Accumulation of 4-HNE can also trigger ferroptosis, airway inflammation, bronchoconstriction, mucus overproduction, and airflow obstruction characteristic of asthma. Collectively, 4-HNE functions as both a mediator of inflammatory injury and a biomarker of oxidative airway damage in asthma. The image was created by using BioRender.com. COM, N. (2026), https://BioRender.com/b7ljcah (accessed on 28 February 2026).

Similarly, oxidative stress plays a major role in the pathophysiology of asthma ([45], Figure 4). Environmental oxidants, infectious agents, and allergens have been shown to generate reactive oxygen species and disturb cellular redox status. Inflammatory cells, including mast cells, eosinophils, neutrophils, macrophages, and lymphocytes, are activated by exposure to pollutants and allergens. Further, activated immune cells release substantial amounts of ROS, which can damage the airway and lung tissues. They can cause lipid peroxidation, producing 4-HNE and other toxic lipid aldehydes that further exacerbate this process. As these free radicals and lipid aldehydes bind to proteins and nucleic acids, altering their structure and function, they can lead to airway remodeling, hyperresponsiveness, mucous production, and airway obstruction [46]. Indeed, several studies have shown increased lipid peroxidation in asthma. For example, Corradi et al. [47] have shown the presence of various lipid peroxidation-derived aldehydes, such as 4-HNE, MDA, 4-hydroxyhexenal, acrolein, n-hexanal, and n-nonanal, in the exhaled breath condensate and induced sputum supernatants of asthma subjects. Similarly, McGovern et al. [48] showed that mice exposed to chlorine (Cl2) developed airway hyperresponsiveness, increased 4-HNE levels, and disrupted redox homeostasis in the lungs. Treatment of mice with the antioxidant dimethylthiourea (DMTU) reduced airway dysfunction and lung protein leakage without altering inflammatory cell recruitment. Specifically, they have shown that DMTU, given before or after Cl2 exposure, prevented 4-HNE accumulation in mice, indicating that 4-HNE is a key marker and mediator of chlorine-induced lipid peroxidation and oxidative lung injury.

Ferroptosis is an iron-dependent form of cell death due to increased oxidative stress and lipid peroxidation [49,50]. Several studies have suggested the significance of ferroptosis in the pathophysiology of asthma and COPD [51]. Wang et al. [52] have shown that ferroptosis is a key mechanism causing neutrophilic airway inflammation in asthma. In this study, they showed increased 4-HNE levels in neutrophilic asthma. Further treatment with quercetin reduced 4-HNE levels, restored antioxidant defenses, suppressed M1 polarization, and airway inflammation. This study also indicates that targeting 4-HNE–linked ferroptosis pathways could be a promising therapeutic strategy in neutrophilic asthma. Similarly, Yu et al. [53] showed that PM2.5 and its component, indeno [1,2,3-cd] pyrene, can enhance oxidative stress and the sensitivity of airway epithelial cells to ferroptosis in a mouse model of asthma. Further, 4-HNE levels were elevated, indicating their role in ferroptosis-driven epithelial injury and airway inflammation. This study indicates that 4-HNE–mediated ferroptosis is a novel therapeutic target in pollution-associated asthma.

Similarly, Li et al. [54] showed that polydatin improved asthma symptoms and lung function in a rat model by reducing iron overload and inhibiting ferroptosis. Further, 4-HNE levels were also significantly reduced by polydatin. These data suggest that targeting 4-HNE–mediated ferroptosis pathways via nuclear receptor coactivator 4 (NCOA4) inhibition may be a promising therapeutic strategy for asthma.

Furthermore, Almolki et al. [55] demonstrated increased 4-HNE adducts in an ovalbumin (OVA)-induced asthma model in guinea pigs. Specifically, they have shown that increased 4-HNE adducts and carbonylated proteins in lung homogenates, as well as heme oxygenase, prevent these oxidative stress markers, as well as mucus hypersecretion and hyperresponsiveness. In another study by Castro et al. [56], increased levels of MDA and 4-HNE were found in BAL fluids from mice infected with respiratory syncytial virus (RSV). They have also shown that treatment with the antioxidant butylated hydroxyanisole prevented RSV-induced increases in aldehyde levels and other symptoms of bronchial asthma.

In similar lines, Nandedkar et al. [57] have shown that OVA treatment to mice modestly increased the 4-HNE Michael’s adducts in the lungs. Further, they have shown that treatment with Ac-DWFKAFYDKVAEKFKEAFNH(2) (4F), an apolipoprotein (apo)A-I mimetic that binds to oxidized lipids and improves HDL function, decreases the formation of 4-HNE adducts and reduces pulmonary inflammation and airway resistance. In addition, Johnson et al. [58] have examined the beneficial effects of alternate-day calorie restriction (ADCR) in overweight asthma patients. The results of this study indicate that oxidative stress markers, including 4-HNE adducts, are elevated in obese asthma patients, and the ADCR dietary program significantly reduced 4-HNE adducts and other respiratory and inflammatory markers. These data suggest that dietary metabolic modulation can improve asthma outcomes, in part, by reducing 4-HNE–mediated oxidative injury and inflammatory signaling. Similarly, Fernandez-Boyanapalli et al. [59] have shown that plasma 4-HNE levels are increased in obese asthmatic patients when compared to nonobese patients.

In another cross-sectional study by Corradi et al. [60], exposure to detergents was examined for its effects on various biomarkers in exhaled breath condensate (EBC) among routine hospital cleaners, who are at increased risk of asthma and rhinitis. They have found that EBC contains increased levels of hydrogen peroxide, ammonium, and 4-HNE. Further, 4-HNE levels correlated with H_2_O_2_, indicating that 4-HNE is a marker of lipid peroxidation and oxidative stress in the airways, even in asymptomatic individuals. Further, Ketema et al. [61] examined 10 urinary phthalate metabolites in 421 children (aged 9–12 years) from the Hokkaido Cohort in Japan. They found that urinary phthalate metabolites in children were associated with allergic symptoms and both T2 (FeNO, eosinophils, IgE) and non-T2 (neutrophils) inflammatory biomarkers. Further, phthalate exposure has been associated with oxidative stress biomarkers, including 4-HNE. This suggests that lipid peroxidation is a mechanistic link between environmental exposure and asthma-related immune responses. Further, these results suggest that 4-HNE–mediated oxidative stress may act as an early upstream trigger contributing to mixed inflammatory asthma phenotypes induced by phthalate exposure. Thus, current evidence suggests that elevated HNE levels increase the likelihood of allergic asthma due to their pathologic effects on inflammation and immune responses.

## 4. Hydroxynonenal in COPD

Chronic Obstructive Pulmonary Disease (COPD) is a progressive airflow limitation disease resulting from impaired lung function due to damaged airways and tissue destruction [62]. It is the third leading cause of death globally and the sixth leading cause in the United States [63]. Emphysema and chronic bronchitis are two conditions also commonly referred to as COPD. Emphysema is classified as a disease affecting the alveoli or interalveolar septa, in which damage reduces elastic recoil [64]. Whereas chronic bronchitis is characterized by continuous inflammation and irritation of the bronchial epithelium, resulting in mucosal hypertrophy and mucus secretion [65].

**Figure 5:**
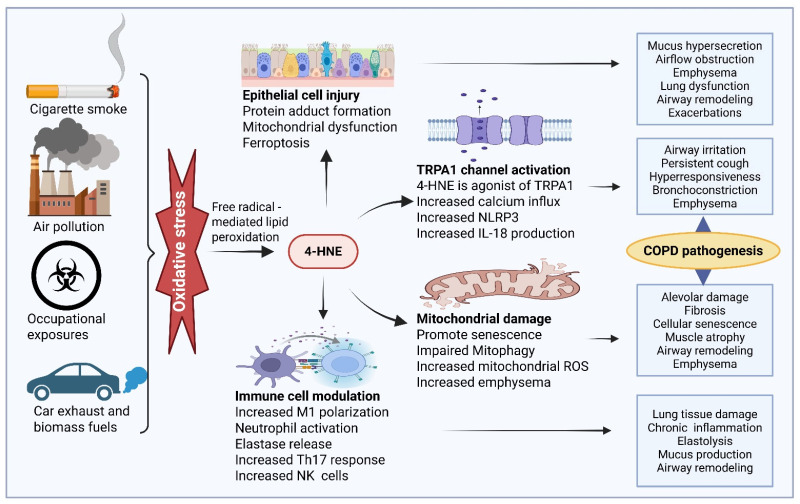
Mechanistic role of 4-hydroxynonenal (4-HNE) in chronic obstructive pulmonary disease (COPD). Chronic exposure to cigarette smoke, air pollutants, and occupational irritants could increase reactive oxygen species (ROS) production. ROS attacks on membrane lipids and causes lipid peroxidation and accumulation of 4-HNE in the airway and alveolar tissues. HNE promotes epithelial injury, mitochondrial dysfunction, immune cell activation, and TRPA1 signaling. These events lead to chronic inflammation, airway remodeling, mucus hypersecretion, and alveolar damage, ultimately contributing to COPD pathogenesis and disease progression. The image was created by using BioRender.com. COM, N. (2026), https://BioRender.com/8mqn871 (accessed on 28 February 2026).

Because COPD results from inflammation due to an immune-mediated response, exposure to pollutants such as noxious particles in vapors, dust, fumes, workplace pollutants, and tobacco smoking is found to be key in the onset of the disease [66]. Tobacco smoking is the leading cause of COPD in the United States, as workplace-related cases mainly go underdiagnosed [67]. Exposure to such pollutants can induce airway damage and inflammation, as well as increase ROS, which initiate oxidative stress responses [68]. As a result of increased ROS from oxidative stress, an accumulation of 4-HNE is found and thought to contribute to COPD [69]. There is a broad consensus that cigarette smoke is the principal risk factor for COPD, as it drives emphysema, chronic inflammation, and oxidative stress central to disease pathogenesis. Several studies have shown that 4-HNE is a possible biomarker for COPD pathology in both preclinical and clinical studies ([69,70], Figure 5).

Indeed, 4-HNE has been shown to be a key mediator of oxidative stress and inflammatory response in cigarette smoke-induced human bronchial epithelial cells. Further, treatment with epigallocatechin gallate (EGCG) significantly reduced lipid peroxidation, 4-HNE formation, and its protein adduct formation [71]. Similarly, acacetin has been shown to prevent cigarette smoke extract-induced injury in human bronchial epithelial cells [72]. In this study, levels of 4-HNE, along with mitochondrial lipid peroxidation, were found to be increased in the smoke extract group compared with the control group. Importantly, treatment with acacetin attenuated these effects, reducing 4-HNE accumulation and lipid peroxidation thereby demonstrating its protective role within the same study [72]. Similarly, Sul et al. [73] showed increased lipid peroxidation products, such as MDA and 4-HNE, as well as increased ferroptosis in human bronchial epithelial cells (BEAS-2B) exposed to cigarette smoke. Furthermore, Mukhopadhyay et al. [74] have demonstrated that transient receptor potential subfamily A member 1 (TRPA1) is functionally expressed in non-neuronal human lung fibroblasts and alveolar epithelial cells, triggering calcium influx and IL-8 release. Most importantly, they have shown that 4-HNE acts as a TRPA1 agonist and TRPA1-mediated IL-8 release. Thus, in COPD, 4-HNE functions not only as a marker of oxidative damage but also as an active mediator that amplifies airway inflammation by activating TRPA1.

In addition, Lanzetti et al. [75] have shown that 4-HNE levels are increased in lung cells of mice exposed to cigarette smoke. Further, treatment with Mate tea significantly reduced 4-HNE levels, inflammatory response, and increased antioxidant defenses. These results indicate that 4-HNE is a key marker of cigarette smoke–induced lipid peroxidation and oxidative lung injury. Similarly, another study by Messier et al. [76] showed that 4-HNE levels are significantly elevated in alveolar type II cells in vitro and in vivo in cigarette smoke-induced Nrf2-deficient mice and wild-type mice. Further, they have shown that N-acetylcysteine protects against pulmonary injury in mice and to prevent the accumulation of 4-HNE. In another study, the same group of researchers reported increased 4-HNE levels in cigarette smoke-induced COPD and their prevention by another antioxidant, Trolox [77].

Another study conducted by Araya et al. [78] showed the significance of 4-HNE in cigarette smoke-induced emphysema, mitochondrial damage, and cellular senescence in COPD mouse models. They have shown that the exposure of cigarette smoke to putative protein kinase 1-deficient mice leads to the accumulation of damaged mitochondria, 4-HNE, and accelerated senescence. However, PRKN overexpression restored mitophagy, reduced mitochondrial ROS and oxidative stress, and 4-HNE levels. This data again suggests that 4-HNE could mediate cigarette smoke -induced mitochondrial oxidative injury in COPD.

Li et al. [79] have shown that 4-HNE plays a major role in the development of COPD in a rat model. They have shown that Recuperating Lung Decoction (RLD), a traditional Chinese medical formula, significantly alleviates oxidative stress in a rat model of COPD by restoring redox balance in lung tissues. Further, the RLD treatment increased GSH levels and reduced 4-HNE and 8-OHdG levels, indicating decreased lipid peroxidation and oxidative DNA damage. This study suggests the antioxidant properties of RLD prevent COPD by reducing the 4-HNE levels. The significance of 4-HNE as a clinically relevant marker of cigarette smoke–induced COPD is further confirmed using a guinea pig model. Further, Paul et al. [80] have shown that exposure of guinea pigs to chronic cigarette smoke increases plasma levels of 4-HNE, 3-nitrotyrosine, and 8-OHdG, along with weight loss and lung inflammation. Further, pharmacological stimulation of soluble guanylate cyclase (sGC) attenuated the cigarette smoke-induced rise in 4-HNE, nitrated proteins, and normalized lung inflammation severity. Similarly, Cardoso et al. [81] have also shown that antioxidant diallyl disulfide also prevents 4-HNE levels in cigarette smoke-induced COPD in mice. A recent study by Wu et al. [82] examined how long-term cigarette smoke exposure promotes glucocorticoid resistance in COPD through 4-HNE. Increased 4-HNE has been shown to suppress HDAC2 and Nrf2 and to reduce MRP1, limiting 4-HNE efflux and resulting in steroid resistance. These results suggest that 4-HNE is a key mediator of glucocorticoid resistance in COPD.

An interesting study by Lin et al. [83] has examined the role of S100A8, an oxidative stress-responsive and anti-inflammatory protein, in alveolar type II cells isolated from smoking and non-smoking organ donors and emphysema patients. They have shown that S100A8 is markedly reduced in alveolar type II cells from smokers and patients with emphysema. Further, reduced S100A8 expression was negatively correlated with increased 4-HNE levels, thus linking increased lipid peroxidation and oxidative stress to alveolar epithelial injury in COPD and therapeutic targeting of S100A8 for COPD. Similarly, lung tissue samples from 19 COPD and 20 control subjects were examined for expression of EC-SOD, fibulin-5, and lipid peroxidation products [84]. The results suggest that MDA is a better lipid aldehyde than 4-HNE for distinguishing COPD patients.

A clinical study by Gifford et al. [85] demonstrated that patients with COPD exhibited persistent skeletal muscle mitochondrial dysfunction, even after matching for physical activity (PA) with controls. In this study, nine COPD patients and nine PA-matched controls were examined. The results show that 4-HNE levels were significantly elevated in the muscle of COPD patients and were associated with reduced mitochondrial density, impaired respiratory and pulmonary function. This study suggests that 4-HNE plays a major role in oxidative stress-mediated mitochondrial dysfunction in COPD skeletal muscle independent of sedentary behavior. In another clinical study by Lu and Cheng [86], it was shown that the levels of 4-HNE, along with IL-16, IL-10, and adiponectin, were increased in the serum and lobectomy lung tissues of COPD patients but not in non-COPD patients.

A case–control study by Barreiro et al. [87] showed that diaphragms from patients with severe COPD show significantly increased protein carbonylation and 4-HNE–protein adducts compared with controls. Further, they showed that, among COPD patients, higher 4-HNE adduct levels were negatively correlated with respiratory muscle strength, linking lipid peroxidation to diaphragmatic dysfunction. The data from this study suggest that 4-HNE is a significant marker of COPD and mediator of oxidative damage, leading to respiratory muscle weakness and disease severity. Similarly, another study by the same group, Barreiro et al. [88], has demonstrated that patients with severe COPD have markedly elevated levels of reactive carbonyls and 4-HNE–protein adducts in the quadriceps. Further, they have shown that elevated 4-HNE levels contribute to baseline muscle dysfunction in COPD. This study also indicates that 4-HNE is a significant COPD marker and mechanistic driver of oxidative muscle injury, linking systemic oxidative stress to skeletal muscle weakness and reduced functional capacity.

Similarly, Rahman et al. [89] examined patients with COPD who are current or previous smokers or who do not have COPD. They found elevated levels of modified 4-HNE protein in alveolar and endothelial cells and increased neutrophils in patients with COPD only. Elevated TGF-β1 protein, along with γ-GCS mRNA levels, were also present in response to the 4-HNE levels. This study indicates that 4-HNE is a key inducer of the proinflammatory response, as it triggers TGF-β1 and γ-GCS production, both of which contribute to inflammation. Another study conducted by Liu and Xu [90] evaluated blood serum levels of 4-HNE, TNF-α, and IL-6 in patients with acute exacerbation of chronic obstructive pulmonary disease (AECOPD) and stable COPD. It was found that there was an increase in both 4-HNE and IL-6 in AECOPD patients compared with stable COPD patients. Recently, Zhang et al. [91] indicated that plasma 4-HNE levels could serve as a biomarker for assessing the severity and prognosis of AECOPD patients. The above-discussed preclinical and clinical studies imply the significance of 4-HNE in the pathophysiology of COPD.

## 5. Hydroxynonenal in ARDS and ALI

Acute lung injury (ALI) and acute respiratory distress syndrome (ARDS) belong to the same group of respiratory diseases, noted by lung inflammation, pulmonary oedema, and hypoxemia to result in acute respiratory failure [92]. Both the infectious and non-infectious conditions have been identified as triggers of ARDS, including sepsis, shock from severe traumatic injuries, blood product transfusions, and smoking [93]. ARDS is present in about 10% of patients in the ICU with a hospital mortality of over 30% [94]. Mortality is more commonly attributed to comorbidities, such as sepsis and multiple organ failure, as it is difficult to determine death from the syndrome itself. Though the Berlin definition is used to classify ARDS severity and mortality (mild, moderate, or severe) in any given individual, a variety of manifestations and responses to treatment in clinical cases make it difficult to study and treat most efficiently [95]. The Berlin definition characterizes ARDS based on acute onset, quantitative arterial hypoxemia, radiographic findings, risk factors, and the exclusion of cardiac causes [96]. Subgroups of ARDS can be identified based on imaging, clinical manifestation, and biomarkers [97]. Recent studies suggest that mild ARDS is also considered as ALI [98].

Currently, there is no specific therapy to treat or prevent ARDS or acute lung injury (ALI) directly [99]. Supportive care via mechanical ventilation remains the key to dealing with this condition. Most cases require invasive ventilation, including sedation and intubation. In most serious cases, extracorporeal membrane oxygenation (ECMO) may be required to remove waste products from the blood and deliver supplemental oxygen [100]. Furthermore, dysregulated lung inflammation is the primary and most significant cause of ARDS. This process is regulated by the binding of endogenous molecules, including ROS, chemokines, and cytokines. Another major factor in the pathogenesis of ARDS/ALI is the increased epithelial and endothelial permeability in the lung tissue [101]. Damage from inflammation disrupts cadherin junctions in the microvascular barrier, leading to the accumulation of alveolar fluid and causing pulmonary oedema [101]. Pulmonary compliance is then decreased, and gas exchange is made difficult, resulting in tissue hypoxia and oxidative stress [102].

**Figure 6:**
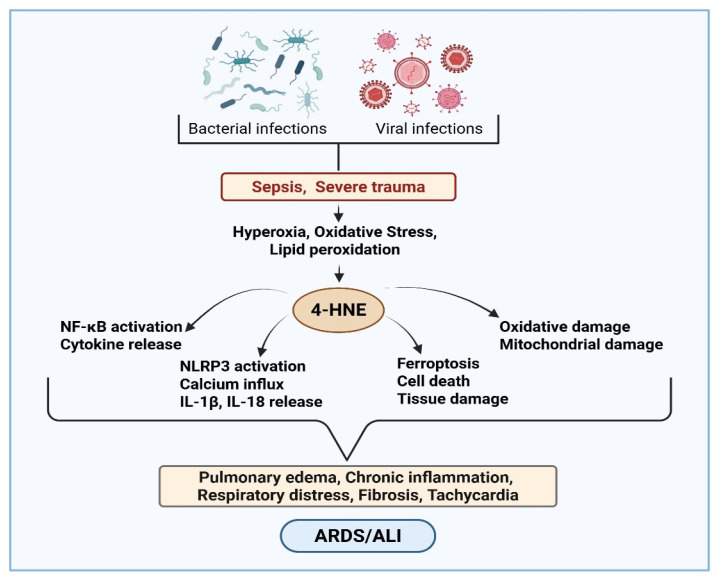
Role of 4-hydroxynonenal (4-HNE) in acute lung injury (ALI) and acute respiratory distress syndrome (ARDS). Sepsis, severe trauma, and infections can cause hyperoxia and oxidative stress. Oxidative stress-generated free radicals cause lipid peroxidation and form 4-HNE in the lungs. HNE further activates NF-κB and NLRP3 signaling, disrupts mitochondrial function, and promotes ferroptosis. These processes amplify inflammation, pulmonary edema, and tissue damage, leading to the progression of ARDS/ALI. The image was created by using BioRender.com. COM, N. (2026), https://BioRender.com/qfz5ujc (accessed on 28 February 2026).

During bacterial infections, oxidative stress plays a role in the pathophysiology of sepsis, which, through the overproduction of ROS and decreased antioxidant enzyme defense, can lead to organ failure and dysfunction [103,104]. Specifically, ROS has been shown to cause endothelial dysfunction, disseminated intravascular coagulation, and thrombosis, which eventually can lead to multiple organ failure in sepsis. Although neutrophils produce free radicals as a first response to pathogen attack, uncontrolled production can lead to tissue damage and dysfunction. Specifically, ROS has been shown to disrupt cellular membranes, induce lipid peroxidation, and form various lipid aldehydes, aggravating the damaging effects of oxidative stress. Several studies indicate that lipid aldehydes play a role in the pathophysiology of ARDS and ALI during infections ([105,106]; Figure 6).

The role of 4-HNE in ALI was investigated by Compton et al. [107]. They have shown that isolated rat lungs perfused with 4-HNE caused significant lung edema, increased elastance, and increased perfusion pressure. These changes were not seen in the control group lungs without 4-HNE perfusion. These results suggest that 4-HNE generated during sepsis could play a causal role in lung injury by impairing lung function. In addition, Carbonell et al. [108] showed that lipopolysaccharide (LPS)-treated rats had significantly increased plasma levels of 4-HNE and MDA and higher lung expression of inducible nitric oxide synthase. Further, glutathione depletion by using phorone decreased these levels. Further, Ebenezer et al. [109] have shown that inhibition of sphingosine-1-phosphate (S1P) lyase prevents histone acetylation and pro-inflammatory cytokine release and suggests that aldehydes generated from S1P degradation could act as epigenetic regulators of inflammatory genes. Specifically, long-chain fatty aldehydes and lipid peroxidation products, such as 4-HNE, were implicated in altering histone acetylation and inflammatory signaling in acute lung injury. Similarly, Mohamed et al. [110] have shown increased lipid aldehydes, such as 4-HNE and MDA, and protein carbonyls in lipopolysaccharide (LPS)-induced lung tissues, as well as treatment with terretonin, isolated from the Aspergillus genus, prevents the production of these lipid aldehydes and LPS-induced lung damage.

Similarly, another study by Ogihara et al. [111] examined lipid peroxidation-generated plasma aldehyde products in premature infants. Infants who later developed chronic lung disease (CLD) had significantly higher levels of aldehydes such as heptanal, 2-nonenal, and 4-HNE when compared on the day of birth to those who did not develop CLD. The results also suggest that elevated concentrations of these aldehydes within the first 24 h were independently associated with CLD development. 4-HNE ≥ 200 nM and 2-nonenal ≥ 150 nM could serve as the strongest early markers of the disease.

Furthermore, Rotta et al. [112] measured oxidative damage at different ventilation strategies in a juvenile rabbit model of acute lung injury. Specifically, this study measured oxidative lung injury by measuring the 4-HNE and MDA levels. Results suggest that ventilation methods such as HFOV, PLV, and HF-PLV significantly improved oxygenation and lung histology while reducing neutrophil infiltration and 4-HNE-associated oxidative injury compared with conventional ventilation. This study also suggests that using a lower perfluorochemical dose in HF-PLV could reduce leukocyte accumulation and limit 4-HNE–related oxidative damage. Similarly, Wang et al. [113] have examined the role of cytochrome P450 1A2 (CYP1A2) in protecting against hyperoxia-induced lung injury. Mice deficient in Cyp1a2 showed greater lung injury and inflammation, as well as increased lipid peroxidation markers, such as 4-HNE-protein adducts and MDA. The data show that increased 4-HNE levels could increase oxidative damage in the absence of CYP1A2, leading to hyperoxic lung injury. Similarly, Zhao et al. [114] reported increased 4-HNE and MDA levels in the lung tissues of a hyperoxic, exacerbated cecal ligation and puncture model in rats. Another study by Hsu et al. [115] showed that 4-HNE inhibits NLRP3 inflammasome activation and pyroptosis in an ALI model of sepsis. Specifically, they have shown that 4-HNE binds with NLRP3 and stops its interaction with NEK7. Similarly, Lv et al. [116] reported that ferroptosis biomarkers, such as 4-HNE and MDA levels, are increased in an LPS-induced ALI model and that treatment with protecting conjugates in tissue regeneration 1 (PCTR1) protected mice from LPS-induced ALI.

A few clinical studies also suggested the significance of 4-HNE as a marker of lung injury. A clinical study by Lichtenstern et al. [117] found that blood serum concentrations of lipid peroxidation products were significantly higher in patients with ARDS than in those with liver failure. This study focused on the concentration of MDA in those affected with the condition. MDA is another product formed during lipid peroxidation, along with 4-HNE. Further, Zarkovic et al. [118] investigated the role of 4-HNE as a biological predictor of the severity, specifically the morbidity, of inflammatory lung conditions in critically ill patients afflicted with COVID-19. They found a significant increase of 4-HNE in the blood plasma from the lungs of deceased patients compared to those who survived. It is suggested that finding the concentration of 4-HNE in patients with inflammatory lung conditions may lead to more personalized, and thus more effective, treatment and a greater ability to deliver an individual’s prognosis. A similar study from the same group [119] conducted an autopsy study to investigate the role of 4-HNE in patients who died from aggressive COVID-19. They found widespread accumulation of 4-HNE in vital organs, including the lungs. The results also suggest that the lungs and brain are most affected, whereas the kidneys showed limited 4-HNE despite injury. Together, these results suggest 4-HNE, alongside SOD2, as a key link between vascular stress, oxidative damage, and immune dysfunction in lethal SARS-CoV-2 infection. An additional study by the same group also suggested that abundant presence of 4-HNE-protein adducts in the lungs depicts the lethal outcome with aggressive COVID-19 [120]. Similarly, Hosseini et al. [121] reported that COVID-19 lungs showed increased oxidative stress, neutrophil infiltration, and excessive neutrophil extracellular trap (NET) formation. Further, they have shown that 4-HNE is strongly colocalized with NETs and correlated with neutrophil accumulation and increased severity of inflammatory lung damage. Thus, the recent literature suggests that 4-HNE could be a likely biomarker in the pathophysiology of ARDS/ALI.

## 6. Hydroxynonenal in Pneumonitis

**Figure 7:**
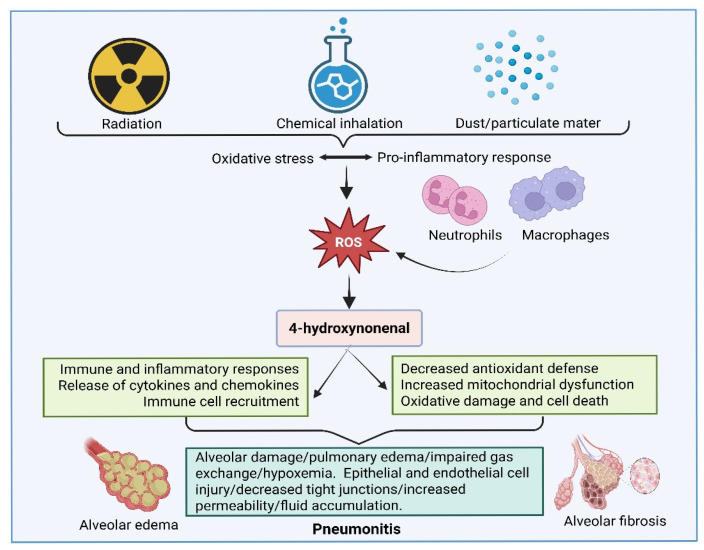
Role of 4-hydroxynonenal (4-HNE) in the pathophysiology of pneumonitis. Radiation- and environmental particulate-induced pneumonitis, as well as hypoxia- and chemical-exposure-induced pneumonitis, involve increased oxidative stress. Oxidative stress leads to the accumulation of lipid peroxidation products, including 4-HNE. The increased 4-HNE in the lungs could impair antioxidant defense and activate inflammatory signaling pathways. These processes can also cause alveolar edema and fibrosis, leading to impaired gas exchange and the progression of pneumonitis. The image was created by using BioRender.com. COM, N. (2026), https://BioRender.com/bfhct8p (accessed on 25 March 2026).

Pneumonitis is characterized by focal or diffuse inflammation of the lung parenchyma. Classification of pneumonitis includes hypersensitivity, radiation, chemical, community-acquired pneumonia, and aspiration pneumonitis. Though the subcategories of pneumonitis differ in causative agents, they share the common symptoms of lung inflammation [122,123,124]. Symptoms in patients with pneumonitis vary from fever, shortness of breath, tachypnea, tachycardia, and altered mental status. Like in many inflammatory complications, oxidative stress is also a triggering factor for pneumonitis [125]. Oxidative stress in pneumonitis is caused by various agents, but during the early phase of injury, increased oxidative stress and ROS induction lead to the production of 4-HNE [126]. A few studies also indicate that 4-HNE mediates signaling pathways and stimulates the expression of inflammatory factors. In turn, this leads to the aggregation of inflammatory responses and causes pneumonitis ([127,128]; Figure 7).

Radiation-induced interstitial pneumonitis in rats showed that oxidative stress caused the greatest lipid peroxidation in lung mitochondria and microsomes. It was also noted that, within the lung cytoplasm, the activities of superoxide dismutase and catalase were reduced in pneumonitis [129]. This reduction in activity may be associated in particular with radiation-induced pneumonitis. Similarly, a recent study by Li et al. [130] showed that Mai-Men-Dong decoction (MMDD) treatment significantly prevented radiation-induced lung injury in mice via the STAT6/p53/SLC7A11 pathway. Further, the levels of 4-HNE and ferroptosis were reduced by MMDD treatment, indicating the significance of lipid peroxidation and oxidative membrane damage in radiation-induced lung injury. In another study by Shvedova et al. [131], exposure of mice to single-walled carbon nanotubes (SWCNTs) resulted in acute lung inflammation, followed by progressive fibrosis, granuloma formation, and impaired respiratory and immune function. Further, SWCNT exposure also led to the accumulation of 4-HNE and depletion of glutathione. This study indicates the significance of 4-HNE in oxidative injury-induced pulmonary inflammation and fibrosis. Similarly, another study by Yanamala et al. [132] examined how exposure to biodiesel (BD) exhaust particles in mice caused greater lung toxicity than petroleum diesel. The results show that, in addition to elevated lung tissue injury markers, BD particulates also led to increased accumulation of oxidatively modified proteins and 4-HNE. Similarly, Natarajan et al. [133] investigated how organic poultry dust extracts induce lung epithelial inflammation through protease activation and increasing the intracellular oxidative stress. They have shown that organic dust induces oxidant production and inflammatory signaling in A549 and Beas2B lung epithelial cells. Specifically, the levels of 4-HNE, along with IL-8, were significantly elevated.

In addition, Francis et al. [134] have shown that ozone exposure triggers pulmonary inflammation, and oxidative lung injury in mice. Further, the splenectomized mice have shown reduced pro-inflammatory macrophage infiltration and chemokine signaling, leading to decreased lung injury. Importantly, 4-HNE levels were also reduced in splenectomized mice, indicating reduced lipid peroxidation and tissue damage. Similarly, Arya et al. [135] demonstrated that cerium oxide nanoparticles protect rat lungs against hypobaric hypoxia-induced lung damage. The prevention of lung damage by these nanoparticles is due to increased glutathione levels, reduced oxidative protein modifications, and reduced 4-HNE adduct formation. These results suggest the significance of lipid peroxidation-derived aldehydes in the hypobaric hypoxia-induced lung inflammation and damage. Furthermore, Schott et al. [136] investigated the link between *Mycoplasma bovis*–associated pneumonia in calves and oxidative and nitrative lung injury. They have shown increased levels of 4-HNE and 3-nitrotyrosine in lung tissues. These results suggest that lipid peroxidation-generated aldehydes within necrotic lesions could contribute to lesion formation and disease severity in *M. bovis* pneumonia.

In a clinical study, Jiang et al. [137] showed that serum 4-HNE levels were significantly elevated in patients with community-acquired pneumonia (CAP) and increased in parallel with disease severity scores. Patients with higher serum 4-HNE concentration on admission have been shown to have augmented mechanical ventilation usage, longer hospital stays, and a higher risk for death during hospitalization. Furthermore, elderly patients with pneumonitis due to CAP were more likely to suffer from the consequences associated with the rise in 4-HNE than younger patients. This study indicates the significance of 4-HNE as a mediator of oxidative lung injury and a powerful early biomarker for disease severity in CAP. Similarly, another clinical study by Wu and Pan [138] indicates that serum 4-HNE levels were significantly higher in patients with severe pneumonia. The results also indicate that elevated 4-HNE levels, along with lactic acid levels, could improve diagnostic reliability and predict poor outcomes. Further, patients who died had markedly higher 4-HNE concentrations, indicating greater oxidative stress and tissue injury. This study also signifies the role of 4-HNE as a biomarker for severe pneumonia.

## 7. Conclusions and Future Perspectives

4-HNE is an essential facilitator of oxidant-induced cell signaling and apoptosis and a product of lipid peroxidation. 4-HNE is a highly reactive electrophilic aldehyde that can diffuse into surrounding tissue cells. 4-HNE then targets lipids, nucleic acids, and proteins, forming adducts that alter cell signaling pathways and cause cellular toxicity. Although 4-HNE shows promise as a biomarker in inflammatory lung disease, current evidence mainly supports its association with disease severity and prognosis rather than its ability to predict disease onset. However, recent studies from the last couple of decades have indicated that 4-HNE could be a potential biomarker of oxidative stress-driven human diseases ([11,139,140]; Table 1). Specifically, through covalent adduct formation and modulation of redox-sensitive signaling, 4-HNE links oxidative stress to inflammatory complications, including various lung-specific inflammatory diseases. As an oxidative stress marker and mediator of injury, 4-HNE has been shown to be involved in the pathophysiology of various lung diseases [141,142,143].

In chronic airway diseases such as asthma and COPD, exposure to allergens, pollutants, and smoke could cause persistent oxidative stress, leading to lipid peroxidation within airway tissues. This leads to the accumulation of 4-HNE in epithelial cells, macrophages, and extracellular matrix components. 4-HNE has been shown to modify NF-κB and NLRP3, as well as alter pro-inflammatory cytokine production and the inflammatory response [11]. Further, several studies have shown that elevated 4-HNE levels in asthma are associated with epithelial injury, mucus hypersecretion, and pulmonary inflammation [50,51,52,53,54,55]. In COPD, chronic accumulation of 4-HNE correlates with emphysematous changes and progressive decline in lung function [70,89,90].

Similarly, in ARDS and ALI, 4-HNE has been shown to play a pivotal role in the transition from acute oxidative injury to sustained inflammation and fibrosis [111,112,113,114,115]. Oxidative stress triggers such as severe infections, mechanical ventilation, and environmental insults in alveolar and endothelial cells cause lipid peroxidation and increased 4-HNE production. In fact, elevated levels of 4-HNE have been detected in bronchoalveolar lavage fluid, plasma, and lung tissue of patients with ALI [89,90]. Several studies have shown that HNE participates in ferroptosis, an iron-dependent form of regulated cell death characterized by lipid peroxidation and membrane damage [144,145,146]. Ferroptosis has been shown to be associated with multiple lung inflammatory complications [147]. For example, accumulation of 4-HNE in bronchial epithelial cells and lung tissues has been identified in the LPS-induced lung injury model [148].

Several studies also indicate that the elevated levels of 4-HNE or 4-HNE-protein adducts are associated with pathological outcomes of several lung inflammatory diseases. Thus, 4-HNE could serve as an important biomarker for determining disease severity and clinical outcomes. In addition, HNE has been shown to regulate multiple signaling pathways that lead to lung inflammatory complications, and targeting 4-HNE formation and metabolism could also offer better therapeutic benefits.

4-HNE is metabolized by enzymes such as GSTs, ALDHs, and AR, which, via conjugation with glutathione, oxidation, or reduction reactions. Thus, therapeutic strategies that modulate 4-HNE metabolism could offer a promising approach in treating lung inflammatory diseases. Indeed, we have shown earlier that AR inhibitors have shown promise in experimental models by suppressing inflammatory signaling pathways activated by 4-HNE and its glutathione conjugates [149,150]. Our previous studies indicate that AR inhibition prevents asthma, COPD, and airway remodeling in mouse models [151,152,153].

Similarly, modulation of ALDHs may influence the balance between detoxification and propagation of oxidative signaling. Generally, these enzymes offer a beneficial role in protecting against lipid aldehyde toxicity. However, activation of these enzymes could be affected by controlling the lipid aldehyde toxicity. Indeed, a few studies indicate that ALDH overexpression protects against oxidant-induced lung injury [154,155]. GST-mediated conjugation of 4-HNE with glutathione represents an additional critical detoxification pathway. Similarly, increasing GST activity may accelerate the clearance of 4-HNE via glutathione conjugation and reduce oxidative damage. It is well known that oxidative stress decreases the levels of cellular glutathione, which are required for the formation of 4-HNE-glutathione conjugates. Further, therapeutic approaches that restore glutathione homeostasis, activate Nrf2-dependent antioxidant pathways, or specifically target GST isoforms may help maintain optimal 4-HNE metabolism and limit tissue injury.

Besides direct modulation of detoxification enzymes, broader strategies to reduce lipid peroxidation could also help control 4-HNE production. Several studies have shown that various antioxidants, ferroptosis inhibitors, and agents that stabilize mitochondrial function could prevent experimental models of lung injury [156,157,158]. Further, nanotechnology-based antioxidant delivery systems should be investigated to reduce the lipid aldehyde-mediated damage to lung tissues.

Additional research is needed to develop more standardized methods for measuring 4-HNE and its protein adducts in various biological samples. More specific and effective methods will establish clinically relevant thresholds that correlate with disease severity and outcomes. There are no large, longitudinal clinical studies conducted to validate the use of 4-HNE as a diagnostic and prognostic biomarker among different lung diseases and patient populations. Such studies are needed to detect disease progression early and guide therapeutic measures. Similarly, studies using recent proteomics and metabolomics could help identify novel pathways altered by 4-HNE. Such studies will also identify potential drug targets for disease intervention. In similar lines, additional studies are needed to understand how 4-HNE interacts with immune cells and lung epithelial cells at different stages of lung injury, which will be essential for developing targeted therapeutics.

Thus, without any doubt, the available literature suggests that 4-HNE could serve as a critical link between oxidative stress and pulmonary inflammation in lung inflammatory complications. Further, a few studies suggest that the 4-HNE’s consistent association with disease severity and outcomes signifies its value as both a biomarker and a mediator of pathophysiology [47,60,83,90,137]. However, despite these promising findings, clinical studies remain limited in number and are largely observational. There is currently insufficient evidence to support the therapeutic targeting of 4-HNE in clinical practice. Thus, the development of novel therapeutic approaches that control 4-HNE metabolism, such as AR inhibitors, will help prevent these complications. However, several translational research studies are necessary to clarify the mechanisms by which these inhibitors regulate 4-HNE metabolism, contribute to lung inflammatory complications, and develop specific therapeutic interventions.

## Figures and Tables

**Table 1:** Evidence supporting the role of 4-HNE as a biomarker in lung inflammatory complications.

Disease	Biological Sample (Model)	Biomarker Evidence	Pathological Significance	Citations
Asthma	Exhaled breath condensate, sputum (Humans)	Increased 4-HNE and other lipid aldehydes	Reflects airway oxidative stress and inflammation	[47]
	Chlorine exposure model (Animals)	Increased 4-HNE associated with airway hyperresponsiveness	Marker of oxidative lung injury	[48]
	Neutrophilic asthma models (Animals)	Increased 4-HNE associated with ferroptosis	Biomarker of inflammatory phenotype	[52,53,54]
	OVA-induced lung tissue (Animals)	Increased 4-HNE–protein adducts	Indicates airway remodeling and inflammation	[55,56,57]
	Serum (Humans; obese/severe asthma)	Increased systemic 4-HNE levels	Associated with disease severity	[58,59]
	Environmental exposure cohorts (Humans)	Increased 4-HNE correlates with oxidative markers	Early biomarker of airway injury	[60]
COPD	Lung tissue, plasma (Humans)	Increased 4-HNE accumulation	Biomarker of lipid peroxidation in COPD	[69,70]
	Bronchial epithelial cells (Cell models)	Increased 4-HNE and protein adducts	Reflects smoke-induced oxidative injury	[71,72,73]
	Cigarette smoke models (Animals)	Increased 4-HNE linked to mitochondrial damage	Marker of disease progression	[73,74,75,76,77,78]
	Plasma, lung tissue (Animals)	Increased 4-HNE correlates with inflammation/emphysema	Indicator of severity	[79,80,81]
	Lung tissue (Humans)	Increased 4-HNE associated with reduced Nrf2/HDAC2	Biomarker of steroid resistance	[83]
	Skeletal muscle/diaphragm (Humans)	Increased 4-HNE–protein adducts	Correlates with muscle dysfunction and severity	[85,86,87,88]
	Serum during exacerbations (Humans)	Increased 4-HNE levels	Prognostic marker of AECOPD	[90,91]
ARDS/ALI	Plasma, lung tissue (Animals; sepsis models)	Increased 4-HNE associated with oxidative lung injury	Marker of lipid peroxidation	[105,106,107,108]
	Ventilation-induced ALI model (Animals)	Increased 4-HNE used to quantify oxidative injury	Reflects severity and response to ventilation	[112]
	Hyperoxia models (Animals)	Increased 4-HNE–protein adducts	Indicator of oxidative lung damage	[113,114]
	LPS-induced ALI (Animals)	Increased 4-HNE with ferroptosis biomarkers	Marker of epithelial injury	[116]
	Plasma (Humans)	Increased circulating 4-HNE levels	Predictor of severity and mortality	[118,119,120,121]
Pneum-onitis	Lung tissue (Radiation models; Animals)	Increased lipid peroxidation and 4-HNE	Marker of oxidative membrane damage	[129,130]
	Particle exposure models (Animals)	Increased 4-HNE accumulation	Reflects oxidative injury and fibrosis risk	[131,132,133]
	Ozone/hypoxia models (Animals)	Increased 4-HNE reduced by antioxidants	Tracks injury severity and treatment response	[134,135]
	Lung tissue (Infectious pneumonitis; Animals)	Increased 4-HNE detected in lesions	Associated with disease severity	[136]
	Serum (Humans; pneumonia/CAP)	Increased serum 4-HNE correlates with severity and mortality	Early prognostic biomarker	[137,138]

## Data Availability

No new data were created or analyzed in this study. Data sharing is not applicable to this article.
